# Effects of Multidisciplinary Team Care on the Survival of Patients with Different Stages of Non-Small Cell Lung Cancer: A National Cohort Study

**DOI:** 10.1371/journal.pone.0126547

**Published:** 2015-05-12

**Authors:** Chien-Chou Pan, Pei-Tseng Kung, Yueh-Hsin Wang, Yu-Chia Chang, Shih-Ting Wang, Wen-Chen Tsai

**Affiliations:** 1 Department of Health Services Administration, China Medical University, Taichung, Taiwan; 2 Department of Public Health, China Medical University, Taichung, Taiwan; 3 Department of Orthopedic Surgery, Taichung Veterans General Hospital, Taichung, Taiwan; 4 Department of Healthcare Administration, Asia University, Taichung, Taiwan; Memorial Sloan-Kettering Cancer Center, UNITED STATES

## Abstract

In Taiwan, cancer is the top cause of death, and the mortality rate of lung cancer is the highest of all cancers. Some studies have demonstrated that multidisciplinary team (MDT) care can improve survival rates of non-small cell lung cancer (NSCLC) patients. However, no study has discussed the effect of MDT care on different stages of NSCLC. The target population for this study consisted of patients with NSCLC newly diagnosed in the 2005–2010 Cancer Registry. The data was linked with the 2002–2011 National Health Insurance Research Database and the 2005–2011 Cause of Death Statistics Database. The multivariate Cox proportional hazards model was used to explore whether the involvement of MDT care had an effect on survival. This study applied the propensity score as a control variable to reduce selection bias between patients with and without involvement of MDT care. The adjusted hazard ratio (HR) of death of MDT participants with stage III & IV NSCLC was significantly lower than that of MDT non-participants (adjusted HR = 0.87, 95% confidence interval = 0.84-0.90). This study revealed that MDT care are significantly associated with higher survival rate of patients with stage III and IV NSCLC, and thus MDT care should be used in the treatment of these patients.

## Introduction

Lung cancer is the most common cause of death among all cancers in humans. Every year, about 1.4 million people die from lung cancer worldwide.[1] According to the report of the World Health Organization, tracheal, bronchus and lung cancers, together, are the 7th leading cause of death, and were the only cancers among the top 10 causes of death in 2011 in the world.[2] The 5-year survival rate of lung cancer ranges from 73% in stage IA to 2% in stage IV, and is about 16% overall.[3] In Taiwan, malignant tumor (cancer) is the top cause of death[4], and the mortality rate of lung cancer is the highest of all cancers.[5]

Treatment of lung cancer is based on different cancer stages. Surgical intervention is usually adequate in stage I non-small cell lung cancer (NSCLC).[6] Radical resection and adjuvant chemotherapy are indicated in stage II NSCLC.[6] In stage IIIA NSCLC, surgery, adjuvant chemotherapy and postoperative radiotherapy are necessary for these patients.[6] In stage IIIB and IV patients, treatment should emphasize palliation of symptoms and improvement of life quality. Because of the diverse cancer stages, treating patients is always a challenge for surgeons, oncologists, radiotherapists, social workers, nurses and many other team members. Compared to the treatment of early-stage cancer patients, treatment for advanced-stage cancer patients is usually more complicated, and thus requires more staffs with different specialties. This becomes a multidisciplinary team (MDT), involving different professional members who cooperate and coordinate tightly to treat patients.

MDT care has been practiced for many years in many countries. It usually includes surgeons, medical oncologists, radiation oncologists, pathologists, cancer care nurses, case coordinators, nutritionists, physiotherapists, psychologists, or social workers. The MDT meetings are held regularly and the members collaborate together to make treatment protocol for each patient. The goal of the MDT care is to provide a complete cancer therapy protocol and thus improve the quality of cancer diagnosis and treatment. MDT care is important in the management of patients with cancer.

In Taiwan, a project called "Cancer Centers for a Great Improvement in the Quality of Cancer Care" was launched by Health Promotion Administration, Ministry of Health and Welfare in 2003.[7] This project was to increase the quality of prevention, diagnosis and treatment of cancer. Several fundamental works were established, and the first one is "organizing a multidisciplinary cancer treatment team". Hospitals which were approved to participate in this project should follow the "Regulations for Cancer Care Quality Assurance Measures". To improve quality of cancer care, a quality control team should be set up under the patient-centered principle. Based on this project, the team leader coordinated the subspecialties and provided adequate treatment and care protocol for the patients. Thus, patients were treated by this integrated MDT, but no longer by individual physicians. In addition to improve quality of care and patient-centered treatment, the database of diagnosis and treatment were set up for further evaluation or research.

MDT project is a demonstration program in Taiwan. This policy encourages hospitals which treat cancer patients to join this program. The MDT should have regular combined conference and discuss the treatment protocol of newly diagnosed cancer patients. Some specific documentation of these patients should be sent to NHI administration. Hospitals can have extra reimbursement, which is $2000 New Taiwan Dollars (about USD 65) per patient, from the NHI Administration. For this reason, in hospitals which have MDT program, physicians usually arrange patients to join the MDT treatment. However, not all hospitals join the project. Two factors affect hospitals to join this program: hospitals which provide service of cancer treatment, and hospitals which are large enough to be able to set up departments of subspecialties to meet the requirement of the program. Most of large hospitals, which are medical centers or regional hospitals, join this program.

MDT care could improve life quality of patients. Ellis et al. found that MDT care can increase quality of life in NSCLC patients[8]. Many papers reported the survival of cancer patients could be improved by implantation of MDT care. Wang et al. found that the relative risk of death of oral cancer patients was lower for MDT participants[9]. Chang et al. found that the overall survival of hepatocellur patients were significantly improved after the establishment of the MDT treatment[10]. Kesson et al. reported that MDT care was associated with increased survival in breast cancer[11]. Morales et al. found that MDT care could increase the resectability and survival rates in pancreatic cancer patients[12]. Forrest et al. reported increased survival of NSCLC patients after implantation of MDT care[13]. Friedland et al. found that MDT care could increase survival of patients with stage IV head and neck cancer[14].

However, there has been no study discussing the effect of MDT care on different stages of NSCLC. This study was designed to analyze the factors affecting survival of patients with NSCLC, and most of all, to find out whether MDT care can result in a different survival rate at each stage of NSCLC.

## Materials and Methods

### Study Subjects

This study was a retrospective and longitudinal study with a nationwide cohort. The target population for this study was patients with lung cancer (International Classification of Disease for Oncology, 3rd edition, ICD-O-3, C339~C349) newly diagnosed in the 2005–2010 Cancer Registry. Those who had received treatment (including surgery, radiotherapy, or chemotherapy) within the first year after diagnosed constituted the study sample. Lymphoma and sarcoma are not tumors which specifically originate in lung. The treatment of small cell lung cancer is mainly by chemotherapy and is different from that of non-small cell lung cancer[15–18]. We excluded patients with the pathologies of lymphoma (ICD-O-3 9590~9989), sarcoma (ICD-O-3 8800~8806), and small cell lung cancer (ICD-O-3 8041, 8043, 8044, and 8045). Patients who suffered from carcinoma in situ, received hospice care only, or had no biopsy diagnosis were also excluded.

### Data Sources

As a retrospective cohort study involving analysis of secondary data, this study used the 2005–2010 "Taiwan Cancer Registry" published by the Taiwan Health Promotion Administration as the basis for selecting the target population. Combined with data from the 2002–2011 “National Health Insurance Research Database” provided by the Taiwan Ministry of Health and Welfare, the health status of the target population before and after development of cancer, as well as the healthcare utilization, treatment methods, and personal traits of patients at the time of cancer diagnosis were analyzed. The 2005–2011 “Cause of Death Statistics Database” was used as criteria for determining whether or not a specific patient had passed away.

### Descriptions of Variables

In this study, residence areas for the population were divided into seven levels by the degree of urbanization in each, with level 1 being the most urbanized and level 7 being the least urbanized [19]. Comorbidity was evaluated by the Charlson Comorbidity Index (CCI) modified and developed by Deyo et al [20]. The scores consisted of CCI 0–3, 4–6, 7–9, and ≧10 points. A higher score would mean a higher level of comorbidity. The annual service volume of hospitals and physicians were combined into the annual service volume for cancer patients under treatment for the specific year. Adopting the similar method published by Yu et al. [21], the service volume was divided by the median value, and was then divided into a high and a low annual service volume. Cancer stages in this study were based on the staging system developed by the American Joint Committee on Cancer (AJCC), where TNM is used to describe the condition of each patient (T: tumor; N: node; M: metastasis) [22]. The data of cancer stage came from 2005–2010 "Taiwan Cancer Registry". The data of participation in MDT care, gender, age, urbanization level of the residence area, premium-based monthly salary, catastrophic injuries or illnesses except for cancer, the annual service volume of the primary healthcare provider and the attending physician came from 2002–2011 "National Health Insurance Research Database". The data of death came from 2005–2011 "Cause of Death Database".

### Statistical Analysis

The Chi-square test was first used to explore whether or not NSCLC patients' participation in MDT care was related to their individual characteristics, including gender, age, urbanization level of the residence area, socioeconomic status (including premium-based monthly salary), health condition (including cancer stage, CCI), presence of other catastrophic injuries or illnesses besides cancer, and the annual service volume of the primary healthcare provider and the attending physician. The propensity score (PS), which has being widely adopted in many papers[9, 23, 24], was used in this study to balance the groups of MDT participants and MDT non-participants groups to reduce selection bias. It was the conditional probability of each MDT participants and its calculation was based on the variables that were listed in Table 1. Using the multivariate logistic regression model, the probability of involving MDT care for different cancer patients was estimated. In total, 2808 MDT participants and 20667 MDT non-participants were included in the study. Then, the multivariate Cox proportional hazards model explored whether the involvement of MDT care had an effect on survival when individual characteristics, socioeconomic status, health condition, cancer stage, and the annual service volume of the primary healthcare provider and the attending physician were controlled. The same method has been used by Tsai et al. and was published in Jan, 2015[25]. In order to reduce selection bias, we placed the probability of MDT involvement of each patient as a control variable in the model. Finally, the multivariate Cox proportional hazards model was used to analyze the influence of a multidisciplinary diagnosis and treatment team on the different stages of cancer, to produce the adjusted Cox survival curve. This model was introduced by Cox[26] and was widely adopted in estimation of survival. Unlike the unadjusted survival curve which was made by using the Kaplan-Meier method, the Cox proportional hazards model can be used to investigate several variables at a time. Statistical significance was defined as p-value < 0.05. All statistical analyses were performed using SAS software (Version 9.3, SAS Institute Inc., Cary, NC) and SPSS (Version 19, IBM SPSS Inc., Chicago, IL).

**Table 1:** Bivariate analysis of patients: MDT participants and non-participants.

Variables		Total		Non-MDT		MDT		P value
		N	%	N	%	N	%	
**Total**		32569	100.00	27937	85.78	4632	14.22	
**Gender**								<0.001
	Female	11536	35.42	9780	84.78	1756	15.22	
	Male	21033	64.58	18157	86.33	2876	13.67	
**Age at diagnosed**								<0.001
	Under 44	1758	5.4	1495	85.04	263	14.96	
	45–54	4666	14.33	3938	84.4	728	15.6	
	55–64	7060	21.68	5964	84.48	1096	15.52	
	65–74	9489	29.14	8127	85.65	1362	14.35	
	Above 75	9596	29.46	8413	87.67	1183	12.33	
**Mean age at diagnosed**		66.13	12.50	66.29	12.51	65.15	12.40	
**CCI score**								<0.001
	0–3	14533	44.62	12333	84.86	2200	15.14	
	4–6	5990	18.39	5152	86.01	838	13.99	
	Above 7	12046	36.99	10452	86.77	1594	13.23	
**Catastrophic illness/injury**								0.100
	Without	31523	96.79	27021	85.72	4502	14.28	
	With	1046	3.21	916	87.57	130	12.43	
**Cancer stage**								0.079
	Stage I	3520	10.81	2981	84.69	539	15.31	
	Stage II	1102	3.38	931	84.48	171	15.52	
	Stage III	9378	28.79	8034	85.67	1344	14.33	
	Stage IV	18569	57.01	15991	86.12	2578	13.88	
**Cancer stage**								0.018
	Stage I+II	4622	14.19	3912	84.64	710	15.36	
	Stage III+IV	27947	85.81	24025	85.97	3922	14.03	
**Hospital level**								<0.001
	Medical center	22293	68.45	20010	89.76	2283	10.24	
	Regional hospital	9739	29.9	7424	76.23	2315	23.77	
	District hospital	537	1.65	503	93.67	34	6.33	
**Hospital ownership**								<0.001
	Public	12706	39.01	11721	92.25	985	7.75	
	Private	19863	60.99	16216	81.64	3647	18.36	
**Service volume of hospital**								<0.001
	Low	1313	4.03	1182	90.02	131	9.98	
	High	31256	95.97	26755	85.6	4501	14.4	

This study was approved by the institutional review board (IRB) of China Medical University and Hospital (IRB number: CMUH102-REC3-076).

## Results

Total subjects in this study were 32,569. The MDT participant and non-participant groups revealed significant differences in gender, age at diagnosed, CCI, the level of hospitals, the ownership of hospitals, the annual service volume of the hospitals, and cancer stage (divided into two groups: stage I&II and stage III&IV). There was no significant difference in catastrophic illness/injury and cancer stage (Table 1).

We built a multivariate logistic regression model that included the following factors: gender, age at diagnosed, CCI, catastrophic illness or injury, level of hospital, ownership of hospital, annual service volume of hospital, and cancer stage. This model was used to predict the probability that patients participated in MDT care; the factors can also be used as variables in the following analysis using the multivariate Cox proportional hazards model. The factors that significantly associated with the probability of MDT participation included patients’ age at diagnosed, CCI score, level of hospital, ownership of hospital, service volume of hospital, and cancer stage. Catastrophic illness/injury had no significant association with the probability of MDT participation (Table 2).

**Table 2:** Factors affecting patients' participation in MDT care by using multivariate logistic regression model.

Variables		OR	95% CI		P value
**Gender**					
	Female ref.)				
	Male	0.92	0.86	0.98	0.013
**Age at diagnosed**					
	Under 44 (ref.)				
	45–54	1.00	0.86	1.17	0.976
	55–64	0.99	0.85	1.15	0.868
	65–74	0.89	0.76	1.03	0.104
	Above 75	0.77	0.66	0.89	0.001
**CCI score**					
	0–3 (ref.)				
	4–6	0.94	0.86	1.02	0.143
	Above 7	0.88	0.82	0.95	0.001
**Catastrophic illness/injury**					
	Without (ref.)				
	With	0.85	0.70	1.03	0.097
**Cancer stage**					
	Stage I+II (ref.)				
	Stage III+IV	0.85	0.77	0.93	<0.001
**Hospital level**					
	Medical center (ref.)				
	Regional hospital	2.36	2.20	2.53	<0.001
	District hospital	1.14	0.77	1.67	0.520
**Hospital ownership**					
	Public (ref.)				
	Private	1.99	1.84	2.16	<0.001
**Service volume of Hospital**					
	Low (ref.)				
	High	2.13	1.74	2.61	<0.001

Event = MDT.

Several factors affected the patients' hazard ratio of death (Table 3). Compared to the reference groups, the variables that had a significantly lower adjusted HR of death were as follows: MDT participant (adjusted HR = 0.49, 95% CI = 0.41–0.57), patients with a premium-based monthly salary of NT 22,801 or more (adjusted HR = 0.79, 95% CI = 0.76–0.82), and patients treated by attending physicians with a high annual service volume (adjusted HR = 0.80, 95% CI = 0.77–0.82). Compared to the reference groups, the variables that had a significantly higher hazard ratio of death were as follows: male patients (adjusted HR = 1.35, 95% CI = 1.31–1.39), patients aged 75 or older (adjusted HR = 1.38, 95% CI = 1.30–1.48), patients residing in level 6 and level 7 urbanized areas (adjusted HR = 1.07, 95% CI = 1.02–1.12), patients treated at regional hospitals (adjusted HR = 2.30, 95% CI = 2.05–2.58) and district hospitals (adjusted HR = 2.05, 95% CI = 1.82–2.31), patients treated at private hospitals (adjusted HR = 1.81, 95% CI = 1.69–1.93), and patients treated at high service-volume hospitals (adjusted HR = 1.69, 95% CI = 1.49–1.90). In addition, the results also showed that adjusted HR of death increased significantly with a higher CCI and advanced cancer stage. The adjusted HR of death for patients with CCI 4~6 and above 10 were 2.09-fold (95% CI = 1.98–2.21) and 2.60-fold (95% CI = 2.49–2.73) that of the patients with CCI 0~3, respectively. We also found that there was interaction between MDT participation and cancer stage. Patients at stage III&IV had a 2.68-fold (95% CI = 2.55–2.82) higher risk of death than patients at stage I&II.

**Table 3:** Factors affecting survival of patients by using multivariate Cox proportional hazards model.

Variables		Adj. HR	95% CI		P value
**MDT status**					
	Non-participants (ref.)				
	Participants	0.49	0.41	0.57	<0.001
**Propensity Score**		0.00	0.00	0.01	<0.001
**Cancer stage**					
	Stage I+II (ref.)				
	Stage III+IV	2.68	2.55	2.82	<0.001
Interaction-MDT*Stage		1.18	1.13	1.24	<0.001
**Gender**					
	Female (ref.)				
	Male	1.35	1.31	1.39	<0.001
**Age at diagnosed**					
	Under 44 (ref.)				
	45–54	0.96	0.91	1.02	0.214
	55–64	0.97	0.92	1.03	0.383
	65–74	1.07	1.01	1.14	0.025
	Above 75	1.38	1.30	1.48	<0.001
**Premium-based monthly salary (NTD)**					
	Under 17280 (ref.)				
	Insured dependent	0.93	0.90	0.97	<0.001
	17281–22800	0.94	0.91	0.97	0.001
	Above 22801	0.79	0.76	0.82	<0.001
**Urbanization of residence area**					
	Level 1 (ref.)				
	Level 2&3	1.00	0.97	1.03	0.905
	Level 4&5	1.03	0.99	1.07	0.149
	Level 6&7	1.07	1.02	1.12	0.008
**CCI score**					
	0–3 (ref.)				
	4–6	2.09	1.98	2.21	<0.001
	Above 7	2.60	2.49	2.73	<0.001
**Catastrophic illness/injury**					
	Without (ref.)				
	With	1.00	0.96	1.05	0.887
**Hospital level**					
	Medical center (ref.)				
	Regional hospital	2.30	2.05	2.58	<0.001
	District hospital	2.05	1.82	2.31	<0.001
**Hospital ownership**					
	Public (ref.)				
	Private	1.81	1.69	1.93	<0.001
**Service volume of hospital**					
	Low (ref.)				
	High	1.69	1.49	1.90	<0.001
**Service volume of attending physicians**					
	Low (ref.)				
	High	0.80	0.77	0.82	<0.001

Event = Death.

**Fig 1:**
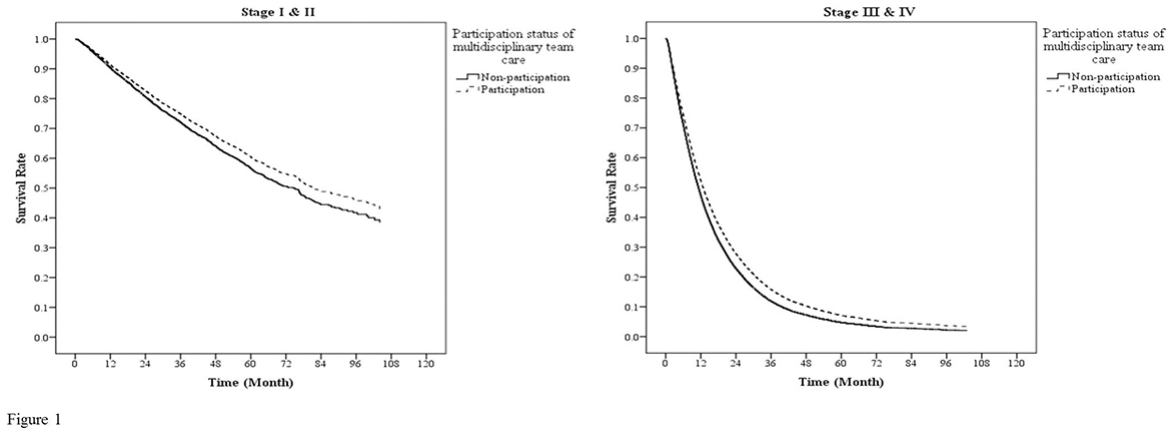
Survival curves of NSCLC patients according to stages. The survival curves were controlled by gender, age, monthly salary, urbanization of residence area, CCI, catastrophic illness, level of hospital, ownership of hospital, annual service volume of hospitals and attending physicians. In stage I&II, there was no statistical significance in the survival rates between MDT participants and MDT non-participants (adjusted HR = 0.89, 95%CI: 0.78–1.01). In stage III&IV, the survival rates of MDT participants were significantly higher than those of MDT non-participants (adjusted HR = 0.87, 95%CI: 0.84–0.90).

Our data showed the 2-year survival rate of MDT participants / non-participants of NSCLC patients were as follows: 81% / 78% (stage I), 64% / 59% (stage II), 37% / 31% (stage III), and 22% / 20% (stage IV). In addition, there was interaction between MDT participation and cancer stage and we found that the significance of association between MDT and risk of death at different stages was not the same. Table 4 showed adjusted HR of death of MDT participants, compared with MDT non-participants, at different cancer stages. Compared to MDT non-participants, the adjusted HR of death of MDT participants was statistically significantly lower among the patients at stage III&IV. In this group, the adjusted HR of death of MDT participants was 0.87-fold (95% CI = 0.84–0.90) that of MDT non-participants. Among patients at stage I&II, the adjusted HR of death of MDT participants was 0.89-fold (95% CI = 0.78–1.01) that of MDT non-participants, but it did not reach statistical significance. These results are shown as the adjusted Cox survival curve (Fig 1).

**Table 4:** MDT care affecting survival of patients at different stages.

Variables	Non-MDT		MDT		Cox model			
	N	%	N	%	Adj. HR*	95% CI		P value
**Stage I&II**	3912	84.64	710	15.36	0.89	0.78	1.01	0.060
**Stage III&IV**	24025	85.97	3922	14.03	0.87	0.84	0.90	<0.001

Event = Death

* The non-MDT group was the reference group.

## Discussion

MDT care for cancer patients has been widely practiced in many hospitals, especially medical centers, for many years. Though Boxer et al[27] found that MDT care does not improve the survival rate of lung cancer patients, Forrest et al[13] and Ellis et al[8] reported that MDT care is associated with higher survival rate of NSCLC patients. Other studies suggested that MDT care is also associated with better survival rate of patients suffering from specific cancers[8–14]. However, only a few studies have discussed the effects of MDT care on the survival rate at specific stages of some cancers. Friedland et al revealed that MDT care is associated with higher survival rate of patients suffering from stage IV head and neck cancer[14]. Chang et al found that the survival rate of MDT participants of stage II hepatocellular cancer is higher than that of MDT non-participants[10]. Though the results of these studies are diverse, we believe that the effects of MDT care on the survival rate of patients at different cancer stages are not the same. This study showed that MDT participants had a higher survival rate than MDT non-participants, and that MDT care was associated with lower mortality rate of patients of stage III and stage IV. Tsai et al. found that the mortality rate of MDT participants was significantly lower than that of MDT non-participants only in patients of stage IV oral cavity cancer [25]. Similar results were also found in our study. Though the HR of death of MDT participants of stage I&II patients is 0.89 and the p-value is closed to 0.05, the data showed non-significant. MDT care was not significantly associated with the survival rate of patients of stage I&II. Probable reasons may include the following.

In stage I NSCLC, surgical intervention is usually enough to treat the patients. Surgeons play important roles and, after surgical intervention, other professionals are not usually involved in the treatment. However, adjuvant chemotherapy or radiotherapy is necessary in advanced stages, thus more staffs should be involved.[6, 15–18] Sometimes another surgery needs to be performed and surgeons with different subspecialties should be involved to treat diverse complications, which occur mostly in patients at advanced stages. For example, NSCLC cancer patients suffering from pathological fracture of bones should be treated by orthopedic surgeons to stabilize the bones; patients suffering from vertebral metastasis might be operated by spine surgeons for relief of neural compression and restoration of spinal stability. For patients at end stages, palliative therapy and hospice care may play important roles if the patients are not able to tolerate any interventional treatment.[28–31] Compared to the treatment of patients at early stages, more professionals should be involved in the treatment of patients at advanced stages, and MDT care then becomes more important. In this situation, MDT care has a much greater effect on the patients at advanced stages.

There are some treatment guidelines for NSCLC that provide standard treatment protocols and algorithms.[15–18, 32, 33] Physicians can follow these guidelines and then treat the cancer patients more efficiently and safely. However, not every patient fits these guidelines, thus the treatment for these patients cannot strictly follow the treatment protocols. This happens more often with patients at advanced stages because their conditions are more complicated. With some patients, physicians will make treatment decisions via a MDT meeting instead of by following the treatment guidelines.[34] In individualizing the treatment protocol for these patients, who are usually more complicated and at advance stages, MDT care can achieve better treatment outcomes. This may be another reason why MDT care has a greater effect on patients at advanced stages.

In the group of age more than 75, these patients had significantly higher adjusted HR of mortality and the lowest probability participating in MDT care. People with advanced age may more likely suffer from chronic diseases other than NSCLC. Diminished immune function and general health condition may also result in poor tolerance of treatment complications in these patients. These might lead to lower survival rate of the elderly. In addition, some old patients or their families are afraid of side effects of some therapies. These patients might refuse aggressive treatment and prefer palliative treatment, and thus have lower probability of joining MDT treatment.

The adjusted HR of death was lower in patients with higher monthly salary. Health care inequalities is often a problem in many countries including Taiwan. Such condition is due to multiple factors, and Forrest et al. found that socioeconomic inequalities in receipt of treatment can significantly decrease survival of lung cancer patients.[35] Vathesatogkit et al. found that cancer patients with higher income had lower mortality rate.[36] Patients with higher income generally may have better financial support, better accessibility of healthy foods and places to exercise, and thus may have higher survival rate.

Some NSCLC patients may also suffer from other chronic diseases, such as diabetes, hypertension, chronic kidney disease (CKD), or coronary artery disease (CAD) and then have higher CCI. These patients may receive different therapies or take lots of drugs to control their underlying diseases. Sometimes they may also get some complications, which result from their underlying diseases and thus they need additional surgeries or medication. These patients may be more probable to experience complications of diseases or treatment, and thus have higher risk of mortality.

Luft et al. found the patients treated by physicians with high service volume had lower risk of mortality.[37] Physicians with high service volume may accumulate their attending experience that helps to improve their knowledge and treatment skill, and thus decrease the mortality rate of their patients.

This study, which was based on a nationwide database, provides strong evidence that MDT participants of NSCLC patients had higher survival rates. No doubt, for the purpose of lowering the mortality rate, MDT care should be used when treating NSCLC patients. Tsai et al. also found that participants of oral cavity cancer patients had higher survival rates.[25] Though there is little evidence about association between MDT care and the survival rate of patients suffering from the other cancers, MDT care might have positive effects on these patients and could be also implanted for improve the survival status. In addition, this study also found that MDT care was associated with higher survival rate of patients at stage III and IV. This result may help the governments allocate their medical resources. If the medical resources are not sufficient to provide full MDT care for all NSCLC patients, emphasizing the use of MDT care for the patients at stage III and IV might be an efficient way to decrease the mortality rates of these patients.

There are some limitations in this study. Though most demographic characteristics were adjusted by the PS method, this data did not include some factors such as smoking, occupation, specific lifestyles or tumor locations. These factors might influence the mortality rates. In addition, we did not further divide these NSCLC patients into different pathologic types. The survival rates of NSCLC patients with different cell types might not be the same. During the treatment course, most patients kept treatment at the same physicians, but few patients might stop treatment or change physicians or change hospitals. The new physicians or hospitals may or may not join the MDT program. However, the reasons why patients change physicians or hospitals cannot be shown in the NHI data base. Whether these patients completed the treatment also cannot be shown in the data base.

## Conclusions

This study revealed that implantation of MDT care is associated with higher survival rate of patients at stage III and IV NSCLC. However, the relationship between MDT care and the survival rate of patients at stage I and II is not significant. In addition to NSCLC, MDT care might also be associated with increase of survival rate of patients who suffer from other cancers. This study could be adopted to find out the relationship between implantation of MDT care and survival of patients who suffer from other cancers.
